# Functional Measures Are Severely Under-Captured in Electronic Health Records

**DOI:** 10.1093/geroni/igaa057.925

**Published:** 2020-12-16

**Authors:** Nicholas Schiltz, Megan Foradori, Andrew Reimer, Mary Dolansky

**Affiliations:** Case Western Reserve University, Cleveland, Ohio, United States

## Abstract

Electronic health records (EHR) data are increasingly used to inform clinical care decisions, assess quality of care, and identify patients at high-risk of poor outcomes (e.g. readmission). Functional measures—including mobility and the ability to perform activities of daily living (ADLs)—are key indicators associated with health-related quality of life and chronic disease management in older adults. The goal of this analysis was to quantify the extent that measures of function are used in a national pool of structured EHR data. We used 2017-2019 data from IBM Watson Health Explorys, representing EHR data from 27 health systems and 360 hospitals nationwide (n=5,224,530 adults age 65 and older). Structured EHR data were mapped to SNOMED-CT codes that identifed six categories of function: mobility, fine motor, gross motor, large muscle, ADLs, and instrumental ADLs. Results indicated that only 3 of the 6 categories were used: ADLs (4.2% of study population), mobility (3.2%), and gross motor skills (2.4%). Fine motor, IADLs, and large muscle function were not recorded in any patients. These results indicate that functional measures appear to be under-reported in structured EHR data when compared to published estimates of the population prevalence. In conclusion, measures of function and mobility remain largely unused in structured EHR data, likely because this information is either not assessed, unavailable for inclusion, or is captured in a non-structured format (e.g. clinical notes). Comprehensive functional measures need to be added to EHRs to assess quality and improve delivery and outcomes in older adult patients.

